# Supplementary material for: Dynamic transcriptomic profiles of zebrafish gills in response to zinc supplementation
Source: BMC Genomics. 2010 Oct 11;11:553. doi: 10.1186/1471-2164-11-553 (PMC3091702; doi:10.1186/1471-2164-11-553)
Supplement: Additional file 2 — Interactive Direct Interaction Network representing the molecular interactions between zinc, copper, iron, calcium and proteins encoded by transcripts changed by zinc supplementation. Mini web-site containing index.html and hyperlinked pages in subdirectory describing a Direct Interaction Network automatically generated based on curated interactions contained within the proprietary PathwayArchitect database. Ovals represent proteins and the circles symbolize metal ions. Objects are coloured by their abundance in zebrafish at the time-point they were significantly different from the control is a scale from -4 fold (dark green) to +4 fold (dark red). Where significant differences were found at more than one time-point, the colour overlay shows expression at the first instance. Dark blue squares denote 'binding', and light blue squares 'expression'; green squares stand for 'regulation', green diamonds for 'metabolism', and green circles for 'promoter binding'. Arrow heads indicate directionality of the interaction where annotated. All nodes and edges can be further interrogated by selecting the relative area of the image. [file 1471-2164-11-553-S2.zip › PathwayArchitect Zn xs DIN/471000.html]

# REGULATION:

|  |  |
| --- | --- |
| Type | REGULATION |
| Effect | Positive |


---

|  |  |
| --- | --- |
| Score | 0 |


---

|  |  |
| --- | --- |
| Reference Count | 15 |


---

|  |  |
| --- | --- |
| Mechanism | Unknown |


---

|  |  |
| --- | --- |
| Reference:0 || Sentence | "Unlike zinc, copper in the serum and that associated with metallothionein showed little response to endotoxin." |
| PMID | 6388345 |
| Year | 1984 |
| Species | Rat |
| Journal | Am J Physiol |
| RefScore | 1 |
| Source | PArchNLP |
  |
|  |  |


---

|  |  |
| --- | --- |
 Reference:1 || Sentence | "Increased MT synthesis in the liver may contribute to copper detoxification; the hypothesis of copper entrapment in enterocytes cannot be confirmed." |
| PMID | 8834384 |
| Year | 1996 |
| Species | Rat |
| Journal | Biol Trace Elem Res |
| RefScore | 1 |
| Source | PArchNLP |
  ||  |  |


---

|  |  |
| --- | --- |
 Reference:2 || Sentence | "All the intestines incubated in the low copper media, regardless of rat zinc status, had the majority of copper associated with MT." |
| PMID | 6822916 |
| Year | 1983 |
| Species | Rat |
| Journal | J Nutr |
| RefScore | 2 |
| Source | PArchNLP |
  ||  |  |


---

|  |  |
| --- | --- |
 Reference:3 || Sentence | "It is suggested that zinc interferes with copper absorption by inducing MT, which sequesters copper in the mucosal cells, making it unavailable for serosal transfer." |
| PMID | 6822916 |
| Year | 1983 |
| Species | Rat |
| Journal | J Nutr |
| RefScore | 1 |
| Source | PArchNLP |
  ||  |  |


---

|  |  |
| --- | --- |
 Reference:4 || Sentence | "In hepatocytes however, MT synthesis resulted in decreasing non-MT-associated copper levels in the cytosol for Cu administrations up to 100 microM." |
| PMID | 8133249 |
| Year | 1994 |
| Species | Rat |
| Journal | J Inorg Biochem |
| RefScore | 0 |
| Source | PArchNLP |
  ||  |  |


---

|  |  |
| --- | --- |
 Reference:5 || Sentence | "The chelating agents induce renal and biliary copper excretion and increased synthesis of metallothionein, which attaches and detoxifies intracellular copper, leading to impaired absorption and binding of excess intracellular copper." |
| PMID | 10378366 |
| Year | 1999 |
| Species | Human |
| Journal | Z Gastroenterol |
| RefScore | 1 |
| Source | PArchNLP |
  ||  |  |


---

|  |  |
| --- | --- |
 Reference:6 || Sentence | "Zinc, by inducing intestinal metallothionein, inhibits absorption of copper from food, and inhibits reabsorption of endogenously secreted copper, thereby producing a negative copper balance in Wilson's disease." |
| PMID | 3886759 |
| Year | 1985 |
| Species | Human |
| Journal | J Am Coll Nutr |
| RefScore | 2 |
| Source | PArchNLP |
  ||  |  |


---

|  |  |
| --- | --- |
 Reference:7 || Sentence | "They promote copper excretion by inducing the synthesis of metallothionein in the intestine, thereby blocking copper absorption from the gut." |
| PMID | 15156602 |
| Year | 2003 |
| Species | Human |
| Journal | Med Sci Monit |
| RefScore | 1 |
| Source | PArchNLP |
  ||  |  |


---

|  |  |
| --- | --- |
 Reference:8 || Sentence | "Metallothionein also appears to participate in metabolic interactions between zinc and copper." |
| PMID | 3303133 |
| Year | 1987 |
| Species | Human |
| Journal | Prog Food Nutr Sci |
| RefScore | 2 |
| Source | PArchNLP |
  ||  |  |


---

|  |  |
| --- | --- |
 Reference:9 || Sentence | "In JIB rats, however, liver copper, MT protein, and MT mRNA were significantly elevated, and a high proportion of the intracellular zinc and copper was associated with MT." |
| PMID | 11090006 |
| Year | 2000 |
| Species | Rat |
| Journal | Am Surg |
| RefScore | 2 |
| Source | PArchNLP |
  ||  |  |


---

|  |  |
| --- | --- |
 Reference:10 || Sentence | "Compared to controls, liver and kidneys of diabetic dams showed an increased concentration of zinc and copper that was associated with metallothionein." |
| PMID | 2484570 |
| Year | 1988 |
| Species | Rat |
| Journal | Biol Trace Elem Res |
| RefScore | 2 |
| Source | PArchNLP |
  ||  |  |


---

|  |  |
| --- | --- |
 Reference:11 || Sentence | "Oral intake of zinc sulphate induces the expression of metallothioneins, enzymes that decrease oxidative stress, and leads to higher serum zinc, and lower copper levels." |
| PMID | 14625013 |
| Year | 2003 |
| Species | Mouse |
|  | Human |
| Journal | Neurosci Lett |
| RefScore | 2 |
| Source | PArchNLP |
  ||  |  |


---

|  |  |
| --- | --- |
 Reference:12 || Sentence | "These findings support our hypothesis that intestinal metallothionein induction mediates decreased copper absorption observed during zinc therapy." |
| PMID | 1517684 |
| Year | 1992 |
| Species | Human |
| Journal | J Lab Clin Med |
| RefScore | 0 |
| Source | PArchNLP |
  ||  |  |


---

|  |  |
| --- | --- |
 Reference:13 || Sentence | "Copper-metallothionein (Cu-MT) and MT-associated copper levels were determined to find a possible correlation between cytosolic copper concentrations and MT as a Cu-detoxifying protein." |
| PMID | 7940570 |
| Year | 1994 |
| Species | Rat |
| Journal | Toxicology |
| RefScore | 1 |
| Source | PArchNLP |
  ||  |  |


---

|  |  |
| --- | --- |
 Reference:14 || Sentence | "Further, intracellular non-MT associated copper levels and levels of GSH and SOD were determined." |
| PMID | 7940570 |
| Year | 1994 |
| Species | Rat |
| Journal | Toxicology |
| RefScore | 0 |
| Source | PArchNLP |
  |


---

|  |  |
| --- | --- |
